# Microtube Array Membrane Encapsulated Cell Therapy: A Novel Platform Technology Solution for Treatment of Alzheimer’s Disease

**DOI:** 10.3390/ijms23126855

**Published:** 2022-06-20

**Authors:** Shu-Mei Chen, Tsung-Chin Hsu, Chee-Ho Chew, Wan-Ting Huang, Amanda Lin Chen, Yung-Feng Lin, Sabiha Eddarkaoui, Luc Buee, Chien-Chung Chen

**Affiliations:** 1 Department of Surgery, Division of Neurosurgery, School of Medicine, College of Medicine, Taipei Medical University, Taipei 11031, Taiwan; chen5319@tmu.edu.tw; 2 Department of Neurosurgery, Taipei Medical University Hospital, Taipei 11031, Taiwan; 3 Taipei Neuroscience Institute, Taipei Medical University, Taipei 11031, Taiwan; 4 Graduate Institute of Biomedical Materials & Tissue Engineering, College of Biomedical Engineering, Taipei Medical University, Taipei 11052, Taiwan; alberthsu1997@gmail.com (T.-C.H.); chchew88@gmail.com (C.-H.C.); sandyhuang@mtamtech.com (W.-T.H.); 5 Department of Biology, University of Washington, Seattle, WA 98195, USA; alc48@uw.edu; 6 School of Medical Laboratory Science and Biotechnology, Taipei Medical University, Taipei 11052, Taiwan; yflin@tmu.edu.tw; 7 Lille Neuroscience & Cognition, Inserm, CHU-Lille, Université de Lille, 59045 Lille, France; sabiha.eddarkaoui@inserm.fr (S.E.); luc.buee@inserm.fr (L.B.); 8 NeuroTMU, Lille International Laboratory, Université de Lille, 59000 Lille, France; 9 International Ph.D. Program for Cell Therapy and Regeneration Medicine, College of Medicine, Taipei Medical University, Taipei 110, Taiwan; 10 The Ph.D. Program for Translational Medicine, Taipei Medical University, Taipei 11052, Taiwan; 11 International Ph.D. Program in Biomedical Engineering, College of Biomedical Engineering, Taipei Medical University, Taipei 110, Taiwan; 12 Ph.D. Program in Biotechnology Research and Development, College of Pharmacy, Taipei Medical University, Taipei 110, Taiwan

**Keywords:** neurodegenerative disease, Alzheimer’s disease (AD), passive immunotherapy, encapsulated cell therapy, microtube array membrane (MTAMs)

## Abstract

Alzheimer’s disease is the most frequent form of dementia in aging population and is presently the world’s sixth largest cause of mortality. With the advancement of therapies, several solutions have been developed such as passive immunotherapy against these misfolded proteins, thereby resulting in the clearance. Within this segment, encapsulated cell therapy (ECT) solutions that utilize antibody releasing cells have been proposed with a multitude of techniques under development. Hence, in this study, we utilized our novel and patented Microtube Array Membranes (MTAMs) as an encapsulating platform system with anti-pTau antibody-secreting hybridoma cells to study the impact of it on Alzheimer’s disease. In vivo results revealed that in the water maze, the mice implanted with hybridoma cell MTAMs intracranially (IN) and subcutaneously (SC) showed improvement in the time spent the goal quadrant and escape latency. In passive avoidance, hybridoma cell loaded MTAMs (IN and SC) performed significantly well in step-through latency. At the end of treatment, animals with hybridoma cell loaded MTAMs had lower phosphorylated tau (pTau) expression than empty MTAMs had. Combining both experimental results unveiled that the clearance of phosphorylated tau might rescue the cognitive impairment associated with AD.

## 1. Introduction

Alzheimer’s disease (AD) is a well-known neurodegenerative disease. The status of a healthy brain gradually worsens over time. Symptoms of Alzheimer’s disease such as dementia start interfering with one’s basic activity and memory in their life span when the disease progress into mild cognitive impairment. Statistics show an astonishing prevalence of Alzheimer’s disease in the United States. It was estimated that 5.8 million Americans of all ages had Alzheimer’s disease dementia in 2019. Of this population, 81% of them were elders over 75 years old [1,2]. Thus, AD impacted tremendously on the economic burden. It cost about $290 billion in public health care in the USA in 2019. Multiple factors contributes to the development of AD, including but not limited to brain tumors and deficiency in vitamin D or B12 [3,4]. Scholars have gradually disclosed the relationship between amyloid-β (Aβ) and tau. They suggest that tau and Aβ were disposed to develop simultaneously [5]. On the other hand, these neurotoxic proteins cause synapse loss, oxidative stress, mitochondrial dysfunction, and neuronal death, eventually, accompanying cognitive decline [6,7,8]. Genetically, the early onset of AD was derived from a hereditary disease. There are three main autosomal dominant mutated genes: APP, PSEN1, and PSEN2. These genes guided an increase in Aβ42: Aβ40 ratio, either a rise in Aβ42 and decline in Aβ40, or an increase in both. As a result, the pathway “amyloidogenic cascade” was unlocked [9]. For late onset cases, Apolipoprotein E (ApoE) and apoE4 isoforms have been identified to be responsible for a late onset of AD [10].

Among the multitude of therapies advancement, encapsulated cell therapy (ECT) is one of the leading proposed strategies for the treatment of AD. The fundamental concept of encapsulated cell therapy derived from the 1950s. This technique encapsulates functional living cells within a semi-permeable membrane. This technique prevents encapsulated cells from coming into contact with the adjacent host’s tissue. This in turn, eliminates the possibility of triggering an immune response which might lead to rejection [11]. The porous membrane confers sufficient protection while ensuring supply of oxygen and nutrients diffuse through for cell proliferation, migration, and differentiation [12,13]. These functional or genetically engineered cells enclosed in the membrane can steadily produce therapeutic substances and specific cellular secretions. In one example, Aure’lien’s team genetically engineered C2C12 mouse myoblasts to release murinized antibodies IgG2a against A oligomers and plaques based on the amyloid cascade hypothesis. C2C12 myoblasts inserted in a PEG hydrogel device (106 cells). In the AD mice model, after subcutaneous implantation (TauPS2APP mice, 5XFAD mice). IgG2a was continually secreted by cells to aid in the clearance of A plaques and to prevent additional tauopathy and tau phosphorylation [14,15]. In another example, Xinglong’s group produced the PLGA with curcumin nanoparticles to prevent Aβ buildup; however, if negative effects occur, reversing them is impossible [16]**.**

Recently, there were numerous immunotherapies for anti-Aβ and tau proceed in clinical trials. For example, DC8E8 antibodies inhibited tau-tau interaction by masking the neuron surface proteoglycans [17]. Now, begin introducing a method to stop the early progress of AD pathology in clinical trials, for example, there were compounds to halt the formation of amyloid precursor protein (APP) and interfere with the production of Aβ peptide. Inhibitors of γ-secretase “LY450139 (semagacestat)” developed in Phase Ⅲ (new therapy vs standard existing therapy), however, it did not alleviate the progress of the disease, but also deteriorate the cognitive function. In addition, the compound “methylene blue (chloride methylthioninium; Rember™, TauRx Therapeutics, Singapore)” completing a Phase Ⅱ (safety and effectiveness) could dissolve paired helical filaments (PHFs) of hyper phosphorylated tau to hinder tau aggregation. It showed improvement of cognitive dysfunction in a behavior test by using a tau-transgenic animal model [18].

Despite the positive results discussed above, ECT systems often fall into one of two categories: macroscale systems with a small diffusion surface area or microscale systems with a large diffusion surface area. As a result, current ECT systems are either macroscale, which is recoverable but has a long diffusion distance from the surface to the cells housed within and a low effective surface area, or micron scale, which is nonrecoverable but has a short diffusion distance (50 microns) and an excellent effective large surface for the diffusion of nutrients, gases, and the target therapeutic product [19]. As a result, in present techniques, the inability to recover the encapsulated cells if the treatment has unfavorable/side effects is a big stumbling barrier. The ability to retrieve ECT equipment would be critical in future therapeutic applications, as it would increase patient biosafety [19,20].

In light of these difficulties, we are working to implement a new type of hollow fibers that we invented internally dubbed Microtube Array Membrane (MTAM) [21]. MTAMs are made up of one-to-one coupled ultra-thin microtube fibers that are organized in an array [22,23,24]. The lumen walls of MTAMs are 100 times thinner (2–3 micron) than those of standard hollow fibers (HFs) as compared to traditional HFs. Furthermore, the capacity to alter the microstructures of MTAMs enables us to use them in a variety of applications, including but not limited to encapsulated cell therapy, anti-cancer drug screening, tissue regeneration, green energy, fermentation, bioreactors, and so on [21,22,23,25,26,27,28,29]. The current study, on the other hand, is focused on the encapsulation of hybridoma within Polysulfone (PSF) MTAMs as a potential ‘middle path’ ECT solution that brings tremendous value to potential future patients by incorporating the ability to be recoverable in the event of side effects; while also providing a short diffusion distance of no more than 30 micron from the surface of the MTAMs, which is well within the 50 micron threshold of nutrient and gas diffusion [30]. When the aforementioned general aspects are paired with its outstanding biocompatibility and trans lumen wall diffusion, it becomes a potentially attractive platform to investigate in ECT systems as a prospective faster, accurate, and convenient effect on AD treatment.

## 2. Results

**Figure 1:**
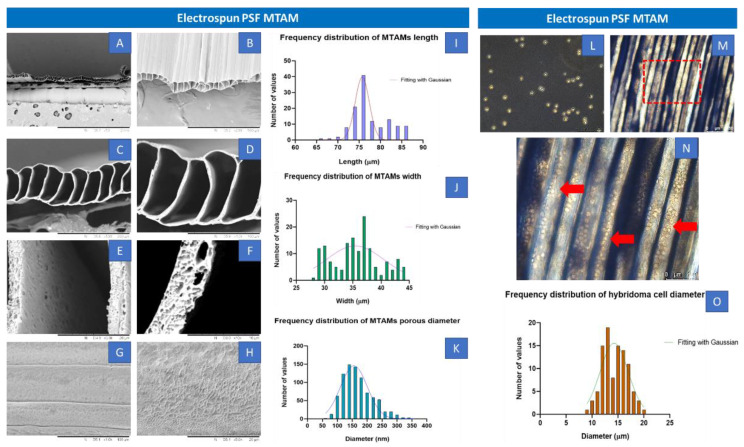
(**A**–**F**) Transverse view of the SEM images of electrospun PSF MTAMs at increasing magnification (50×, 200×, 500×, 1000×, 5000× and 10,000×) and (**G**,**H**) Top view of the SEM images at a magnification 1000× and 5000×. The lumen dimensions of the electrospun PSF MTAMs were about 77.54 ± 4.3 µm × 35.64 ± 4.2 µm (height × width), with a lumen wall thickness of about 4.70 ± 0.3 µm, and a pore size of 167.75 ± 50 nm. (**I**–**K**) Distribution of the MTAM length, width and pore size. (**L**) Optical microscopy images of the hybridoma cells cultured on TCPs, as reference. (**M**,**N**) Optical microscopy images of the PSF MTAMs loaded with hybridoma. (**O**) Distribution size of the hybridoma that were loaded in the PSF MTAMs which averages around 14.4 ± 0.4 µm in diameter (*n* = 6).

Image J quantification of the Scanning Electron Microscopy (SEM) images of the electrospun PSF MTAMs revealed a mean lumen dimension of 77.54 ± 4.3 µm × 35.64 ± 4.2 µm (height × width). The lumen wall thickness of the individual lumen was 4.70 ± 0.3 µm, while the pore size that were detected were around 167.75 ± 50 nm (Figure 1A–K). Majority of the distribution of these measurements were well within the Gaussian distribution curve. As for the hybridoma, those cultured within the standard TCPs that were used as a reference registered a dimension 14.42 ± 0.4 µm, and this size was well within those observed in previous works [23]. These TCP cultured hybridoma proliferated well and the PSF MTAMs of 0.5 cm x 1 cm could easily accommodate the entire hybridoma cell suspension of 2 × 10^5^ cells per 10 μL [23,31]. Once siphoned into the respective PSF MTAMs, the cells could easily be observed by adjusting the focus of the optical microscopy (Figure 1M,N). The siphoned hybridoma appeared to be evenly distributed across multiple individual lumens of the PSF MTAMs.

**Figure 2:**
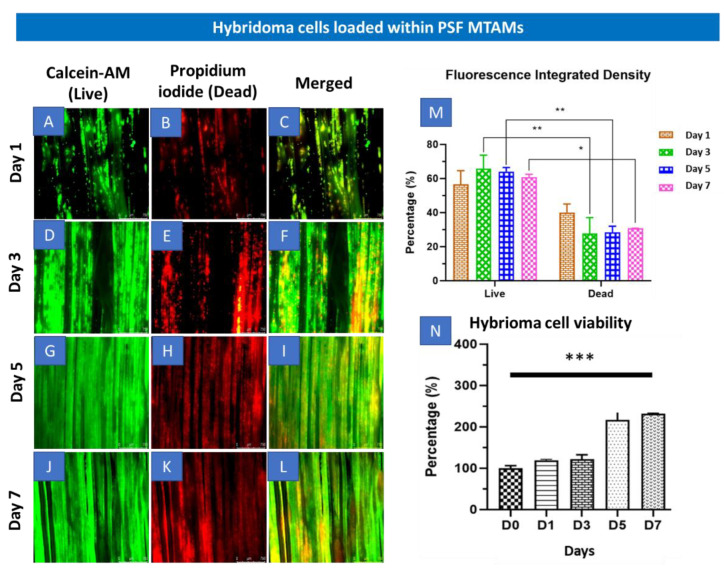
(**A**–**L**) Fluorescence microscopy (4× magnification) of Live-Dead stain of hybridoma cells loaded within PSF MTAMs at day 1 (**A**–**C**), day 3 (**D**–**F**), day 5 (**G**–**I**) and day 7 (**J**–**L**). (**M**) Total fluorescence density of hybridoma cells loaded within the PSF MTAMs at day 7, which revealed significantly higher live hybridoma cells than the dead hybridoma cells. (**N**) Overall viability of hybridoma cells assayed with MTT assay which revealed statistically significant increase between the readings on day 0 versus the viability on day 7. Two-way ANOVA with Sidak’s multiple comparisons tests. * *p* < 0.05, ** *p* < 0.01, *** *p* < 0.001. Error bars in data represented +/−SD (*n* = 3).

With the passage of time, the cell proliferation progressed and began to plane off by day 5 (Figure 2N). By day 7, the overall hybridoma cell viability was statistically significantly higher than that at day 1. These observations were also corroborated in the Live-Dead Stain, where significant increase in cell density appeared between day 3 to 5 (Figure 2D–I). Day 7 fluorescence integrated density also revealed a statistically significant higher live hybridoma (Figure 2M). Additionally, during the staining process, the respective dyes were able to be easily diffused into the lumens of the via highly porous surface, as illustrated in Figure 1, H and also as described in previous works [23,31,32].

**Figure 3:**
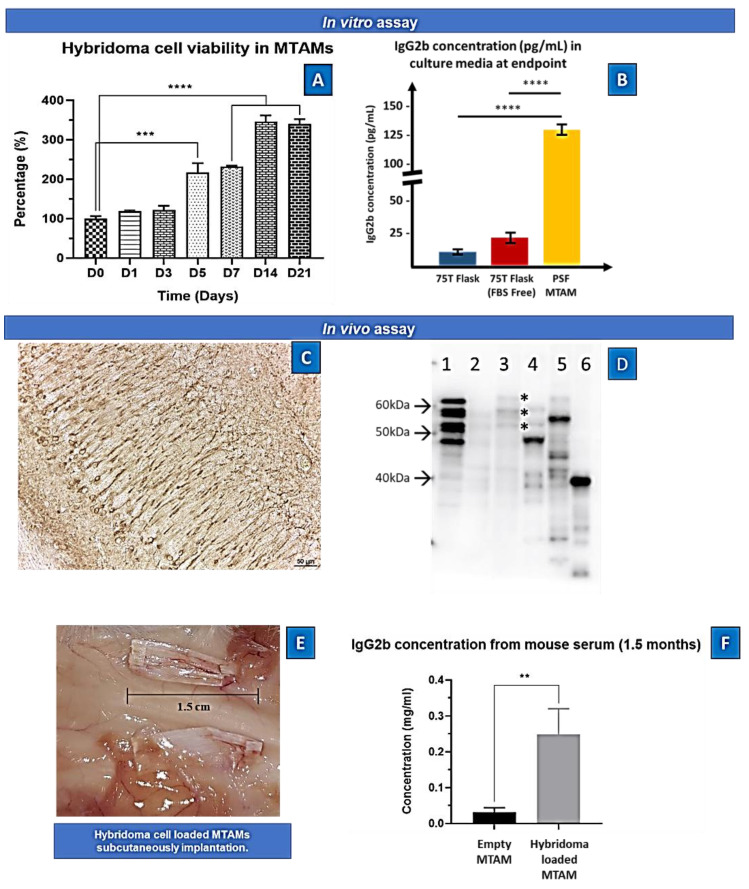
(**A**) In vitro cell viability of hybridoma cells loaded within PSF MTAMs quantified via MTT assay. At day 0, the cell density loaded within the PSF MTAMs were 2 × 10^4^/10 µL. At day 7, 14 and 21 the registered viability increased to 231 ± 5%, 346 ± 26%, and 340 ± 21%, respectively. (**B**) Concentration of IgG2b antibody detected within the culture medium of various culture settings at day 21. One-way ANOVA with Tukey’s multiple comparisons tests: Significant impact *p*-value ** *p* < 0.01, *** *p* < 0.001, **** *p* < 0.0001. Error bars in data represented +/−SD. (*n* = 6) (**C**) Hybridoma supernatant (anti-tau IgG2b)-immunoreactivity in the pyramidale layer of the hippocampus in human tau (1N4R) transgenic mice. Note the strong immunoreactivity in neurons and neurites. Scale: 50 µm. (**D**) Immunoblotting using the hybridoma supernatant (anti-tau IgG2b) on different protein samples: 1. six recombinant tau isoforms (0N3R, 1N3R; 0N4R; 1N4R; 2N3R; 2N4R); 2: human healthy control brain homogenate; 3: Alzheimer’s disease patient brain homogenate; note the typical tau triplet (asterisks); 4: mouse brain homogenate; 5: human tau (1N4R) transgenic mouse brain homogenate; 6: lysate of SY5Y neuroblastoma cell transfected with a fragment of tau protein (amino acids 1–265) indicating that the epitope is in the amino-terminal part of the protein. Molecular weight (arrows) is given on the left of the blot. (**E**) Images of the implanted PSF MTAM loaded with hybridoma cells. (**F**) In vivo assay of the IgG2b antibody at day 90. Animal model with PSF MTAMs loaded with hybridoma cells registered a concentration of 0.25 ± 0.07 mg/mL, which was statistically significantly greater than the animal model with the empty PSF MTAMs.

Both the short term (7 days, Figure 2N) and long term (21 days, Figure 3A) viability of the hybridoma cells encapsulated within the PSF MTAMs revealed significant increase in terms of viability. When compared to day 0, the viability of the hybridoma cells increased to 231 ± 5% and 340 ± 21% respectively; and this represents a statistically significant increase in terms of the overall viability. Interestingly, at day 21 the functional release of antibodies (IgG2b) of hybridoma cells encapsulated within the PSF MTAMs registered significantly higher levels than those cultured within the standard 75T flasks (Figure 3B). This number accounted for a 16-fold increase in functional antibody release of hybridoma cultured within PSF MTAMs as opposed to those cultured within the 75T flask.

In another aspect of this study, the ability of the hybridoma loaded PSF MTAMs were also assessed for its ability to be implanted subcutaneously. Interestingly, there were significantly difference in the serum of the host whereby, the IgG2b antibody levels where the mouse model with hybridoma loaded PSF MTAMs implanted registered an 8.3 fold higher levels of antibody compared to those implanted with empty PSF MTAMs (Figure 3C,D).

In the Morris Water Maze (MWM) test, the baseline of the escape latency for the wild-type C57BL/6J mice was 16 ± 15 s while 3xTg mice registered an escape latency of 14 ± 10 s. For the travel time in goal quadrant, wild-type C57BL/6J mice registered a reading of 22 ± 14 s compared to the 3xTg mice which registered a reading of 35 ± 11 s.

**Figure 4:**
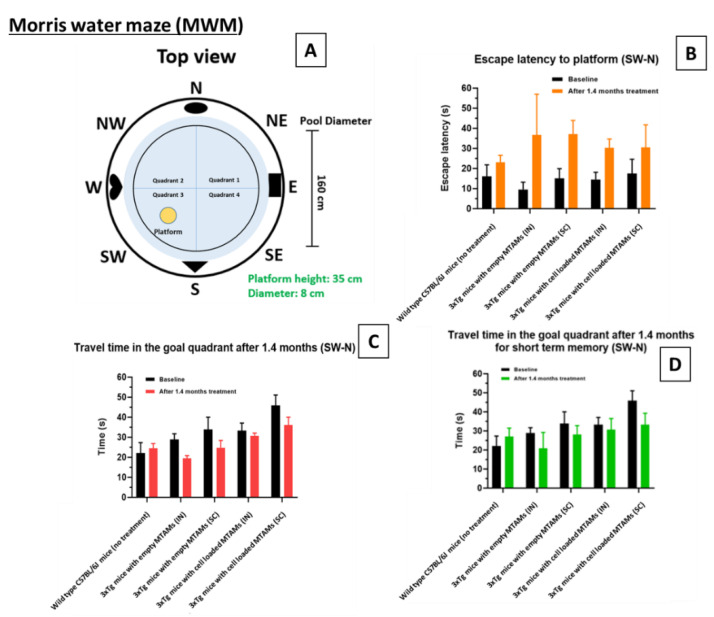
Morris water maze escape latency of the respective models. (**A**) Design of the Morris water maze. The respective study groups were assessed with this system, namely wild-type (without treatment), intracranially (IN) implanted empty PSF MTAMs, subcutaneously (SC) implanted empty PSF MTAMs, IN implanted hybridoma loaded PSF MTAMs, and SC implanted hybridoma loaded PSF MTAMs. (**B**) Escape latency of the respective mouse model study groups analyzed with Two-way ANOVA Tukey’s multiple comparisons tests (Empty MTAMs vs cell loaded: IN group *p*-value = 0.37; SC group *p*-value = 0.58). (**C**) Travel time in the goal quadrant (long term memory testing): mouse models of the study groups with IN and SC implanted with hybridoma PSF MTAMs registered a value of 33 ± 11 s and 46 ± 10 s respectively (baseline). After 1.4 months, this value reduced to 31 ± 4 s and 36 ± 8 s respectively. (**D**) Travel time in the goal quadrant (short term memory testing): the mouse models receiving hybridoma cell loaded PSF MTAMs that were implanted IN and SC registered a reading of 30 ± 12 s and 31 ± 22 s respectively, after 1.4 months of treatment.

When we assessed the long-term memory in the next probing trial, 3xTg mouse for both implant sites (IN and SC) registered a travel time at goal quadrant of 29 ± 6 s and 34 ± 12 s, respectively (Figure 4C). When hybridoma cell loaded PSF MTAMs are implanted instead, a reduction in travel time was observed, registering a reading of 19 ± 3 s (IN) and 25 ± 7 s (SC). After 1.4 months of treatment, the hybridoma cell loaded MTAMs intracranial implantation spent 31 ± 4 s, which was close to baseline at 33 ± 11 s, though the time spent in the goal quadrant for hybridoma cell loaded MTAMs subcutaneous implantation decreased from 46 ± 10 s at baseline to 36 ± 8 s after treatment. In terms of the mice short term memory (Figure 4D), the escape latency of the 3xTg mice models implanted with cell loaded hybridoma PSF MTAMs were 30 ± 12 s (IN) and 31 ± 22 s (SC), respectively. This was significantly lower than those implanted with empty PSF MTAMs respectively. Nonetheless, for short-term memory after 1.4 months, hybridoma cell loaded MTAMs intracranial implantation spent 31 ± 17 s in the goal quadrant, which was higher than empty MTAMs intracranial implantation (21 ± 17 s). The difference between hybridoma cell loaded MTAMs subcutaneous implantation (33 ± 12 s) and empty MTAMs subcutaneous implantation (28 ± 9 s) was not as clear.

Conditioning test is one of the most widely used paradigms to assess learning and memory. In acquisition, firstly, the mice were allowed to explore in the two compartments connected. After exploration, the mice were likely to stay in the dark compartment instead of the light compartment. Next, the mice were given electric foot shock. If the mice remember that danger, the mice will rather stay in the light compartment than move to the dark side.

**Figure 5:**
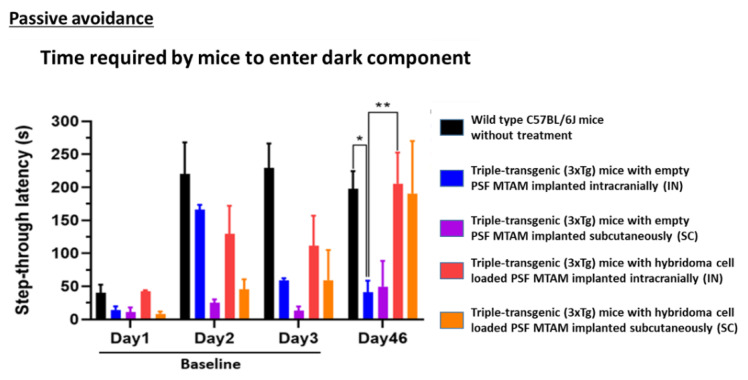
Passive avoidance test mouse models of day 1 to day 3 (baseline) and day 46 of the respective study groups. On day 3, wild-type mouse models registered a step through latency of 229 ± 84 s when compared to the 3xTg mouse with empty PSF MTAM implanted which registered a reading of 61 ± 103 s. At day 46, a statistically significantly different step through latency was observed when comparing the 3xTg mouse with empty PSF MTAM implanted and those implanted with hybridoma cell loaded MTAMs implanted; registering a reading of, intracranial implantation: 41 ± 39 s; vs 205 ± 124 s and subcutaneous implantation 50 ± 55 s vs 190 ± 112 s respectively (3xTg mouse implanted with empty MTAMs versus those implanted with cell loaded MTAMs). Two-way ANOVA with Tukey’s multiple comparisons tests. Significant impact *p*-value * *p* < 0.05, ** *p* < 0.01. Error bars in data represented +/−SD. Wild-type, *n* = 7, empty MTAMs IN and SC, *n* = 5 and *n* = 2; hybridoma cell loaded MTAMs IN and SC, *n* = 7 and *n* = 2.

In Figure 5, on day 3, the time wild-type mice entered the dark compartment was 229 ± 84 s, yet the time 3XTg mice entered the dark compartment was 61 ± 103 s (baseline). After 1.5 months, on day 46, the step-through latency of hybridoma cell loaded MTAMs intracranial implantation and subcutaneous implantation was 205 ± 124 s and 190 ± 112 s, respectively. Nevertheless, the step-through latency of empty MTAMs intracranial implantation and subcutaneous implantation was 41 ± 39 and 50 ± 55 s. To ensure the reproducibility and reliability of results, we tested the empty MTAMs and hybridoma cell loaded MTAMs on further days (Day 46–48). Similarly, we gained the equivalent trend, comparing to the result of day 46. All the time units presented Mean ± SD.

**Figure 6:**
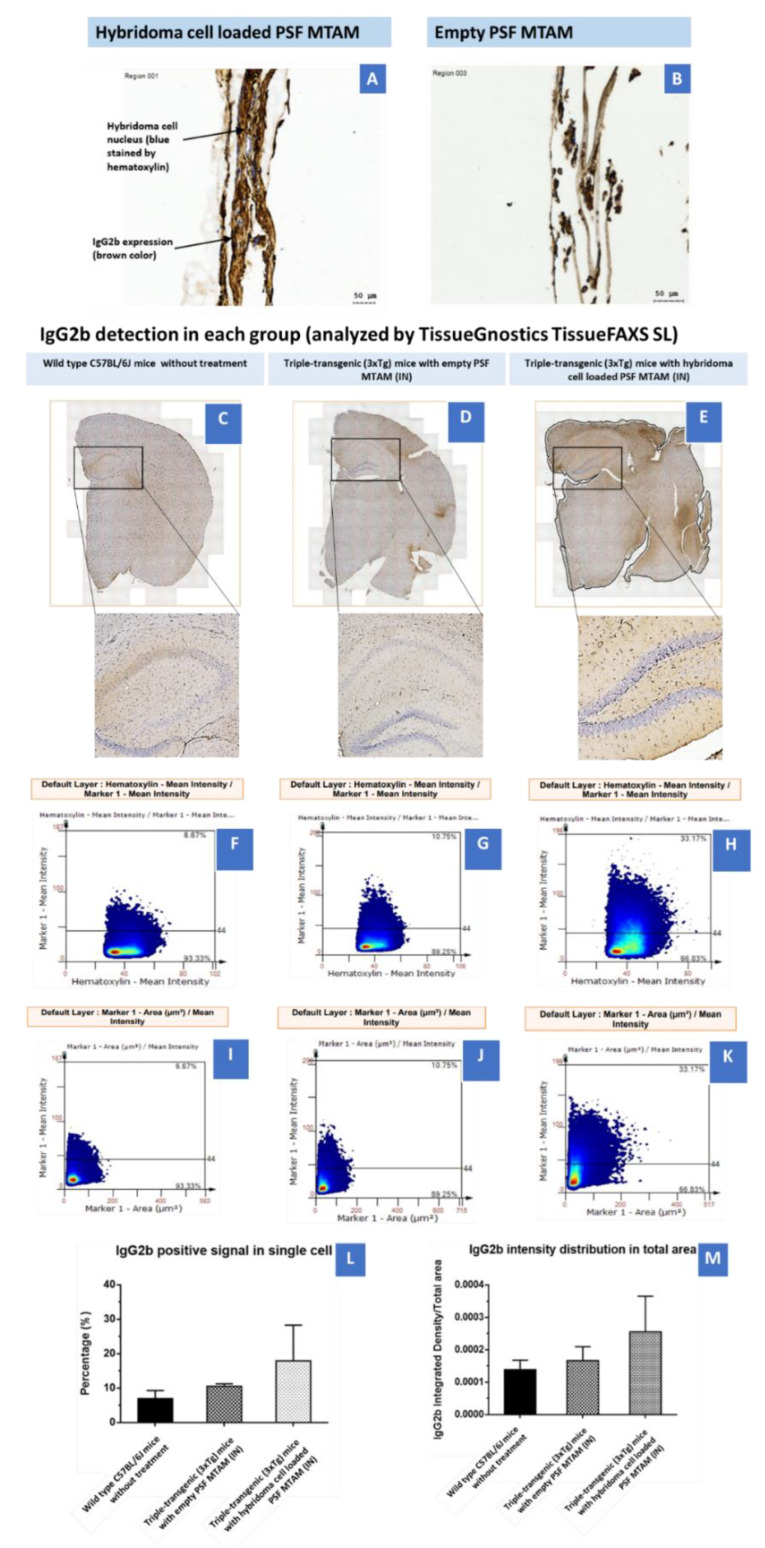
(**A**) Immunohistochemistry of hybridoma cell loaded PSF MTAMs. The nucleus of the hybridoma cells were stained blue from the hematoxylin dye, while the IgG2b antibodies were stained brown. (**B**) Contrary to (**A**), the lack of blue/brown staining were observed within the empty PSF MTAMs. (**C**–**E**) Tissue section of the brain of mouse models of; (**C**) Wild-type C57BL/6J mice without treatment (*n* = 5), (**D**) Triple-transgenic (3xTg) mice with empty PSF MTAM (IN; *n* = 3), and (**E**) Triple-transgenic (3xTg) mice with hybridoma cell loaded PSF MTAM (IN; *n* = 4), along with the corresponding magnified images. IgG2b antibodies appeared to be brown within nucleus, and blue when not within nucleus (counterstained by hematoxylin dye). (**F**–**H**) Percentage of IgG2b positive signal in a single cell (y-axis) with hematoxylin signal (x-axis); and (**I**–**K**) percentages of IgG2b positive signal in a single cell per IgG2b positive area. (**L**) Graph depicting the IgG2b positive signal in a single cell with the Triple-transgenic (3xTg) mice with hybridoma cell loaded PSF MTAM (IN) registering the highest percentage of 18 ± 10% as opposed to those in the wild-type C57BL/6J mice without treatment with a percentage of 7 ± 2%; and (**M**) The corresponding IgG2b antibody distribution in total area.

In the immunohistochemistry staining of the PSF MTAMs (empty and hybridoma cell loaded, Figure 6A,B). The hybridoma cell loaded MTAMs had a firm structure with hybridoma cells entrapped in MTAMs. Moreover, the IgG2b expression was strong in the hybridoma cell loaded MTAMs. The hybridoma cell’s nucleus was stained by hematoxylin. However, the overall structure of empty MTAMs appeared sparse. A weak IgG2b signal could be seen in empty MTAMs. There were no live cells inside the empty MTAMs. To clarify whether IgG2b diffused into cortex and hippocampus by treating hybridoma cell loaded MTAMs intracranially implanted directly on the brain surface, IHC was performed. IgG2b anti-tau antibodies were released by hybridoma cells, which were entrapped and proliferate in a microtube array membrane. The results showed hybridoma cell loaded MTAMs intracranial implantation, empty MTAMs intracranial implantation, and wild-type mice had 18±10, 11±0.7, and 7±2% IgG2b positive area, respectively.

**Figure 7:**
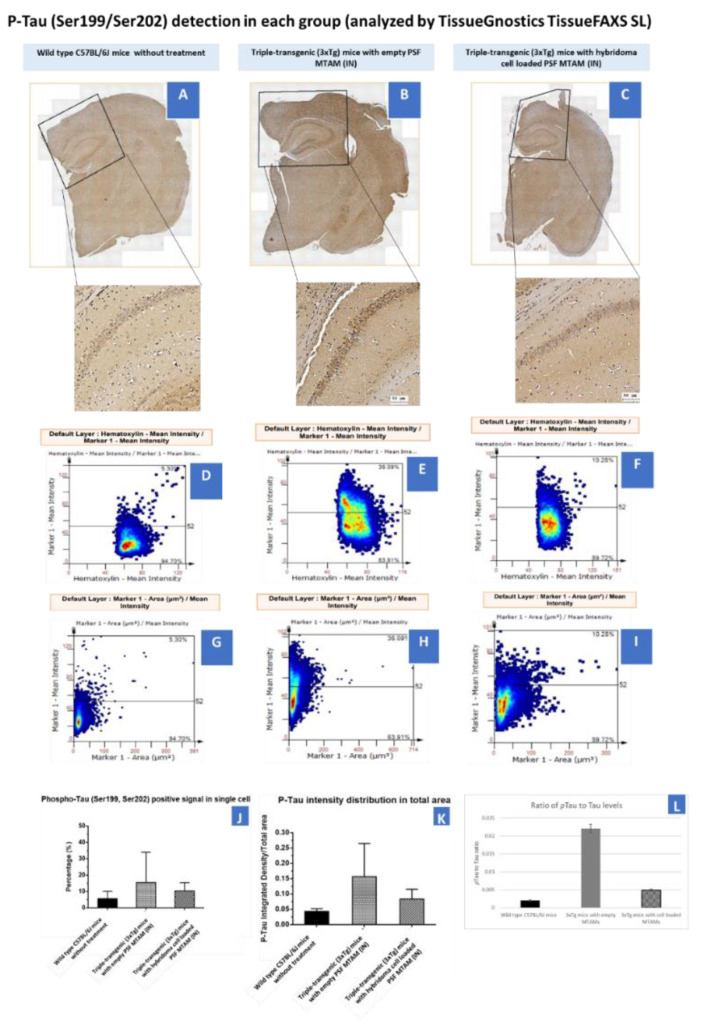
(**A**–**C**) Tissue section of the brain of mouse models of wild-type C57BL/6J mice without treatment (*n* = 5), Triple-transgenic (3xTg) mice with empty PSF MTAM (IN; *n* = 3), and Triple-transgenic (3xTg) mice with hybridoma cell loaded PSF MTAM (IN; *n* = 4) along with the corresponding magnified sections. P-Tau (detected via Ser199/Ser202 antibodies) appeared to be brown in color. (**D**–**F**) Percentage of P-Tau positive signal in a single cell (y-axis) with hematoxylin signal (x-axis); and (**G**–**I**) percentages of P-Tau positive signal in a single cell per P-Tau positive area. (**J**) Graph depicting the P-Tau positive signal in a single cell with the Triple-transgenic (3xTg) mice with empty PSF MTAM (IN) registering the highest percentage of 15 ± 11% as opposed to those in the wild-type C57BL/6J mice (6 ± 3%) and Triple-transgenic (3xTg) mice with hybridoma cell loaded PSF MTAM (IN) (10 ± 3%) respectively. (**K**) The corresponding IgG2b antibody distribution in total area. (**L**) The total Tau levels of the respective study groups.

In determining the P-Tau (Ser199/Ser202) on hippocampus and cortex (Figure 7), hybridoma cell loaded MTAMs intracranial implantation, empty MTAMs intracranial implantation, and wild-type mice had 10 ± 3, 15 ± 11, and 6 ± 3% P-tau positive area, respectively. Previously, half of the brain tissues were on the IHC purpose. For the rest of the half was first to snap freeze by liquid nitrogen and then store at −80 °C for western blot. Hybridoma cell loaded MTAMs intracranial implantation had the ratio of P-Tau and loading control (Beta-actin) in the cortex and hippocampus are 0.41 ± 0.23 and 0.24 ± 0.14, respectively. Empty MTAMs intracranial implantation had the ratio of P-Tau and loading control in cortex and hippocampus are 0.48 ± 0.17 and 0.51 ± 0.11, respectively. Wild-type mice had the ratio of P-Tau and loading control in cortex and hippocampus are 0.15 ± 0 and 0.16 ± 0.06, respectively (Figure 7).

## 3. Discussion

PSF MTAMs were successfully electrospun with the desired microstructures as reported in previous works [23,29,33]. The porous structure was observed in the morphology of MTAMs (Figure 1A–H). It suggested that the effect of blending PSF with Polyvinylpyrrolidone (PVP) exhibited phase inversion [34,35]. Seeding the hybridoma cells in MTAMs revealed that the MTAMs was an excellent shelter to avoid rejection of immune response and can benefit anti-tau IgG2b antibody production, and this was consistent with previously reported findings [23]. Briefly, this was primarily due to the sufficiently small pores which limited physical interaction between the host immune systems and the hybridoma cells encapsulated within, while allowing for nutrients, waste and signaling to pass freely across the lumen wall [32]. Additionally, the semi translucent nature of the electrospun PSF MTAMs allowed for the hybridoma cells encapsulated within to be easily observed via an optical microscope (Figure 1M,N). As the hybridoma cells encapsulated within are potentially overlapping (when observed from above), the fine tuning of focus allowed us to adjust the plane of observation as illustrated in the above-mentioned figure.

In Figure 2, the overall survival of the hybridoma cells loaded within the PSF MTAMs revealed a significant increase between day 0 and day 7 (Figure 2N). Despite being significantly smaller than the standard 75T flask, the ~3× increase in viability seemed to indicate that the PSF MTAM is an excellent substrate for cell culture in part due to the biocompatible nature of the material used; the nano-topography which is conferred by the nano-pores; and the overall 3D configuration which allowed for cell-to-cell interactions as described previously [23,27,36,37]. The increase in viability continued to day 14 and this viability was maintained all the way to day 21, as illustrated in Figure 3A. In terms of antibody released from the hybridoma cell encapsulated within the MTAMs (Figure 3B), the amount of antibody released was significantly higher than those observed in the standard 75T flask. This observation was in line with the previously reported where the microstructure and the close contact between hybridoma cells which allowed for cell-to-cell interaction does indeed trigger significantly higher functional release of antibody [23].

Next, we examined the diffusion of the released antibody into the blood of the host. Indeed, as predicted the hybridoma loaded MTAMs when implanted revealed significantly higher levels of IgG2b antibody levels within the serum, when compared to those implanted with the empty MTAM (Figure 3D). This reinforced the notion that the highly, nano-porous surface of the MTAMs revealed for the diffusion of nutrient, waste and signaling molecules (antibodies in this case), as reported in previous works [32]. While the diffusion of the antibodies across the lumen walls of the PSF MTAMs were not an issue, questions remained on the diffusion of these antibodies across the blood–brain barrier, which indirect evidence provided in Figure 4 will be used for further discussion below.

In the Morris water maze (Figure 4), the 3xTg mice with hybridoma cell loaded PSF MTAMs implanted (IN and SC) registered a significantly longer travel time in the goal quadrant and with shorter escape latency, when compared to those study groups with empty PSF MTAMs implanted. This suggested that those with hybridoma cell loaded PSF MTAMs implanted did indeed registered an improvement in spatial learning and memory abilities of these transgenic mice models [38].

Passive avoidance test which is used to determine the learning and memory of the mouse model; at day 46, the 3xTg mice implanted with hybridoma cell loaded PSF MTAMs registered a statistically significantly higher step through latency when compared against those mouse models implanted with empty PSF MTAMs (Figure 5). This value was almost similar to those observed in the C57BL/6J mice models. This suggested that the hybridoma cell loaded PSF MTAMs does indeed significantly impact the outcome of Tau deposition in the corresponding area of the brain by releasing sufficient IgG2b antibodies, which also reduces neuro-inflammation, and ultimately the memory and learning of these hosts [39,40].

It should also be noted that the behavioral test of the respective mouse models (Figure 5 and Figure 6) suggested that when comparing the performance of those with hybridoma cell loaded PSF MTAMs implanted in the IN site revealed significantly better performance than those implanted on the SC site. While it is tempting to developed medical solutions that are easily applied clinically (i.e., a potential encapsulated cell therapy that is SC implanted that is much more acceptable to patients when compared against brain surgery), we chose at this stage of the development to focus on maximizing the impact of this technology under development. Nevertheless, it should be noted that the SC implantation method does indeed work for delivering therapeutic anti p-Tau antibodies in suppressing the progression of AD (Supplementary Figure S2). Hence, further studies from this point on was conducted on the IN site.

**Figure 8:**
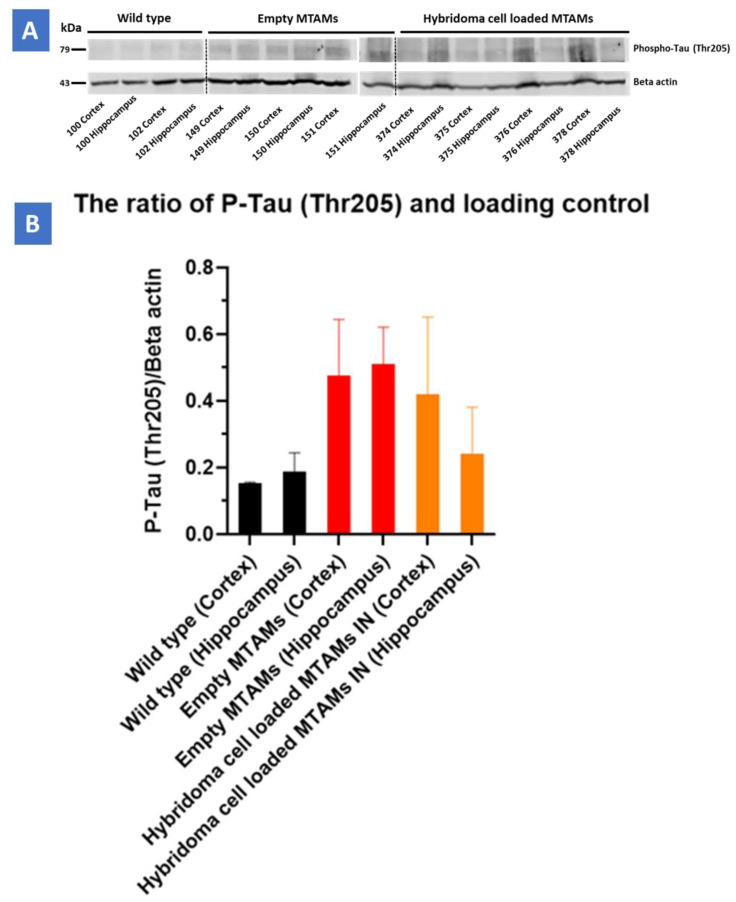
(**A**) Western blot of the respective study groups of the cortex and hippocampus regions targeting the P-Tau protein (79 kDa) and beta-actin (49 kDa). (**B**) Triple-transgenic (3xTg) mice with hybridoma cell loaded PSF MTAM (IN) registered a P-Tau/Beta Actin loading control value at 0.41 ± 0.23 (cortex) and 0.24 ± 0.14 (hippocampus), respectively. Conversely, Triple-transgenic (3xTg) mice with empty PSF MTAM (IN) study group registered the highest values at 0.48 ± 0.17 (cortex) and 0.51 ± 0.11 (hippocampus), respectively, while the wild-type C57BL/6J mice without treatment registered the lowest corresponding values at 0.15 ± 0 (cortex) and 0.16 ± 0.06 (hippocampus).

In Figure 6A,B, the immunostaining of post implantation of the hybridoma cell loaded PSF MTAM and the empty PSF MTAMs revealed significantly distinctive levels of IgG2b antibodies (brown staining) in and around the implanted hybridoma cell loaded PSF MTAMs (Figure 6A). This finding reinforces the notion that these antibodies can freely diffuse from the hybridoma cells encapsulated within the PSF MTAMs to the surrounding tissues, blood stream and ultimately the target site-the brain of the mouse model. Furthermore, the tissue cross section analysis of the antibody deposition in the various regions of the host model’s brains (Figure 6C–K) does further support the above-mentioned notion. Finally, the quantification of the IgG2b antibody in the brain of the mouse model revealed significantly higher levels for those study groups with hybridoma cell loaded PSF MTAMs (Figure 8); and this does indeed suggest the diffusion of the IgG2b antibodies to the respective regions of the brain.

Finally, in regard to the distribution of the Tau protein in the mouse model’s brain (Figure 7), it appeared that regardless of the tissue section of the brain or the final quantification of the Tau protein levels of the mouse model’s brain, an inversely correlated trend between the levels on IgG2b antibodies (Figure 6) and the p-Tau protein levels (Figure 7) was observed. Furthermore, a significant reduction in p-Tau protein levels were observed in those mouse models implanted with hybridoma cell loaded PSF MTAMs (IN) compared to those models implanted with empty PSF MTAMs (Figure 7J,K); and that these reduced levels were close to those observed in the wild-type mice. This suggested that the effect of the antibodies secreted by the encapsulated PSF MTAMs does indeed impact the overall distribution, deposition, and levels of p-Tau protein within the mouse model which ultimately positively impacted the memory, learning and behavioral patterns outline in the above-mentioned tests. In terms of the ratio of pTau to Tau levels (Figure 7L), it was found that the wild-type group registered the lowest reading, while the 3xTg group with empty MTAM implanted registered the highest ratio. Such findings were in line with works by several groups which suggested that the underlying pathology of Alzheimer’s disease is the dysregulation of the balance between the *p*Tau and normal Tau [41]. Conversely, when 3xTg groups were implanted with the hybridoma loaded MTAM, a significant reduction in the ratio was observed, thereby suggesting that the intervention with MTAM loaded with hybridoma cells restore a certain degree of this delicate balance.

## 4. Materials and Methods

### 4.1. Electrospinning of PSF MTAMs

PSF beads (Sigma-Aldrich, Taipei, Taiwan) and polyethylene glycol (Sigma-Aldrich, Taipei, Taiwan) were mixed until homogenous in a 7:3 mixture of N,N-dimethyl formamide (DMF; Tedia, Fairfield, OH, USA) and dichloromethane (DCM; Mallinckrodt, St. Louis, MO, USA). Under ambient conditions, the resulting polymer solution was electrospun as a ‘shell solution’ with a ‘core solution’ comprised of polyethylene glycol (Sigma-Aldrich, St. Louis, MO, USA) and polyethylene oxide (Sigma-Aldrich, St. Louis, MO, USA, as described in previous works [22,25,34]. Briefly, the high voltage charge supply (You-Shang Co., Fongshan City, Taiwan) generated electrostatic force was set up at 4.5–7 kV also current sustained at 750 µA. The distance between the stainless-steel Co-axial spinneret and the rotating drum collector was 50 mm. The rotating drum collector spun at the speed of 100 ± 10 rpm (radius: 70 mm, 0.73 ± 0.07 m/s). After that, the PSF MTAMs were extracted and washed in double distilled water (ddH_2_O), then air-dried. Using a scanning electron microscope, the microstructure characteristics of the PSF MTAMs were measured (SEM; Hitachi, Chiyoda City, Japan).

### 4.2. PSF MTAMs as a Culture Substrate for Hybridoma Cells

Mice were immunized with recombinant human Tau protein as previously described [42]. Briefly, cells from the spleen were fused with myeloma cells to obtain an hybridoma that produced antibodies. Anti-Tau antibody secreting hybridoma cells were isolated to obtain a single clone which was amplified. Hybridoma supernatant contains a monoclonal anti-tau IgG2b antibody which recognizes all tau proteins by immunoblotting and neurofibrillary tangles in tau transgenic mice by immunohistochemistry. In the present work, we used these hybridoma cells. In 50 mL conical tubes, hybridoma cells were centrifuged at 1200 rpm for 5 min. The pellets were collected and suspended in DMEM media at a density of 2105 cells/10 L. The supernatant was discarded, and the pellets were collected and suspended in DMEM media at a density of 2 × 10^5^ cells/10 µL. After that, the relevant PSF MTAMs were used to siphon 10 µL of cell suspension that had been pre-sterilized with UV radiation and diced into 0.5 cm2.0 cm pieces. In order to prepare culture medium, 85% DMEM-high glucose which contained 4500 mg/L glucose and L-glutamine, 3.7 g sodium bicarbonate, 15% percent FBS (HyClone characterized Fetal Bovine Serum, U.S. Origin and 1% PSA (Penicillin/Streptomycin/Amphotericin B (GeneDireX, Inc., New Delhi, India) were prepared and mixed accordingly. Next, hybridoma cells were suspended in freshly prepared medium and incubated at 37 °C in a standard 75T flask, and electrospun PSF MTAMs that were precut into dimensions of 0.5 cm × 2.0 cm under 5% CO_2_ atmosphere, as described in previous work [23]. After the predetermined duration, the respective hybridoma were retrieved and centrifuged at 250× *g* for 5 min to separate the supernatant which was used for antibody quantification and the pallet for viability assessment via MTT assay (3-(4,5-dimethylthiazol-2-yl)-2,5-diphenyltetrazolium bromide) [43].

At the predetermined time points, the respective hybridoma cell loaded PSF MTAMs were retrieved and stained with calcien-AM (Live cells; Biolegend, San Diego, CA, USA) and propidium iodide (Dead cells; Biolegend), incubated for an hour in the dark and immediately visualized under a fluorescent microscope.

### 4.3. IgG2b Antibody Quantification

In the antibody quantification, the supernatant from the abovementioned first centrifugation was transferred into a Amicon^®^ Ultra-15 (30 kDa) Centrifugal Filter (tube) and centrifuged at 7500× *g* at 25 °C for 15 min. The concentrated antibodies were suspended accordingly. Next, 8 points of standard twice diluted in series with standard diluent (the last is blank) were prepared. In each well, add 100 µL standard and samples were transferred and secured with a plate sealer, which was followed by an hour of incubation at 37 °C. Any excessive liquids were removed and 100 µL of biotin-conjugated IgG2b antibody (Detection Reagent A) was added and reincubated for an hour at 37 °C. This process was followed by a wash with a 0.05%Tween-20 in PBS followed by decanting and drying. Next, 100 µL of avidin conjugated to Horseradish Peroxidase (HRP) (Detection Reagent B) was added and incubated for 30 min at 37 °C. Finally, 90 µL TMB substrate was added to each well (turn blue) and left for a 10–20 min reaction time. The reaction was terminated by adding 50 µL stop solution (sulfuric acid) to each well and assayed at 450 nm wavelength in an ELISA reader.

### 4.4. In Vivo Assay

#### 4.4.1. Mice Models and Implant of PSF MTAMs

In this section, Wild-type C57BL/6J mice (female, 2 months old) and Triple-transgenic (3xTg; female, 2 months old) mice inherited the PS1_M146V_, AβPP_swe_, and tau_P301L_ transgenes (Jackson Laboratory, Bar Harbor, ME, USA) were the design of the experiment. All the mice were housed in the standard cage and regularly fed on water and food under temperature and humidity-controlled with the 12 h. light/12 h dark cycle. All the protocols, procedures, and surgery on the animal were accepted by the Institutional Animal Care and Use Committee or Panel (IACUC/IACUP) of Taipei Medical University. Approval No: LAC-2021-0027.

**Figure 9:**
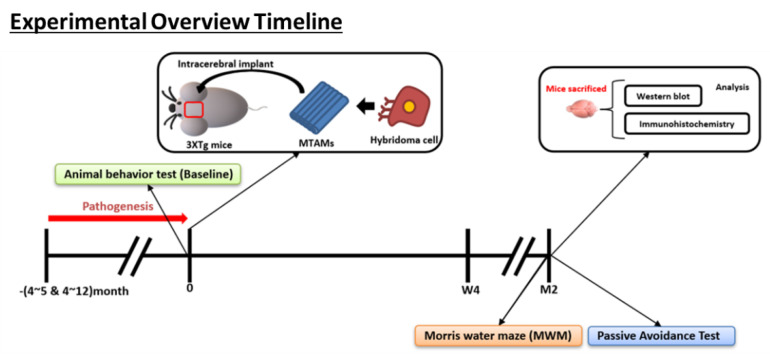
Overview of the animal model timeline. (**Bottom**) Overview on the memory timeline leading up to the Morris water maze for the examination of the short term and long term memory of the respective mice model.

The hybridoma cells were grown as described above (Figure 9) before being siphoned into the PSF MTAMs at a cell density of 2 × 10^5^ cells/10 µL and the ends impulse sealed. The cell-loaded PSF MTAMs were then grown in outlined DMEM medium for 24 h at 37 °C in a 5% CO_2_ environment. For subcutaneous (SC) implantations, the respective mice were anesthetized with xylazine/zoletil mixture (1:1) diluted 10 times by ddH_2_O and administrated through intraperitoneal (IP) injection. The mice’s back fur was shaved and sterilized with 75% alcohol. Next, 0.5 cm longitudinal incisions were made as well as separated mucus layer from the underlying flesh by surgery scissors. Cell loaded MTAMs were well-placed on laboratory spatulas and then implanted subcutaneously. The incisions were sutured and laid on the warm pad until the body temperature recovery before being returned to their respective housings. In the case of intracranial (IN) implantation, the procedure involved were modified based on works by other groups [44]. Briefly, the respective hemisphere of the brain of the mouse models were subjected to craniotomy (three alternating swipes of 70% alcohol and betadine). Minimal dura matter was excised and PSF MTAMs were implanted at the site of excision. The respective bone plates were replaced and secured, and the wound site was closed with sutures.

#### 4.4.2. Assessment/Indicators of the Impact of the Implantation of PSF MTAMs in Mice Models

##### Animal Behavior Test

Morris Water Maze (MWM)

**Figure 10:**
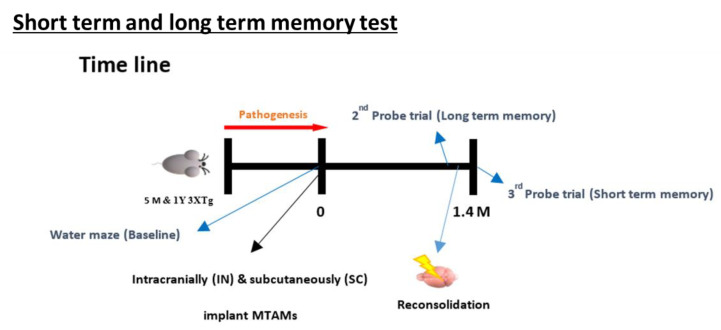
Timeline leading up to the test of the short-term and long-term memory test of the MWM. The procedure involves the training of the mice from day 1–6 based on distal spatial cues to locate the platform where the mice are placed in different random quadrant for it to search for the platform; assessed by the escape latency and time in quadrants in which submerged platform is located in. With these values as the baseline, a probe test was carried out at 1.4 months later where the submerged platform was removed and the above-mentioned tests were repeated. Within 24 h of conducting the long-term memory test, a reconsolidation process was carried out by replacing the submerged platform and repeating the steps outlined in the baseline section. After 24 h, another probe test was carried out again by removing the submerged. The escape latency and time in quadrant at this time will form the basis for the short-term memory [48].

A circular pool with white bottom measuring 150 cm in diameter with a depth of 50 cm was prepared accordingly. Next, 4 high contrast spatial signs were marker on the respective sections of the pool wall (East, West, North, and South). A platform was placed in the target quadrants at a height of 1.5 cm above the water line. Memory acquisition training (Figure 10) was carried out by transferring preadapted mouse models into the respective regions of the pools, and directional guidance toward the platform was provided (in cases where the mouse models were unable to find the platform within a minute); the mouse was allowed to stay on the platform for 15 s. Between day 2–5, the pool was immersed with milk powder to a height of 2 cm above the platform. Mouse models were given 1 min to find the platform. By day 6, the platform was removed, and similar to the steps outlined during the training sessions, the respective mouse models were given 2 min to explore the pool without the platform attached [45,46,47]. All the parameters were recorded and analyzed by ActualTrack: Animal Behavior Analysis Software (A-M System, Sequim, WA, USA).

##### Passive Avoidance Test

A passive avoidance box (PACS_091120, Columbus Instrument) had 2 compartments (light and dark) connecting with a passage was procured. Mouse of all study groups were allowed to separately adapt to the environment within the box on day 1 for at least 30 min. Next, the respective mouse models were transferred into the dark region of the box (gate closed) and allowed to explore for 5 min. This process was repeated with the light region and the respective mouse models were allowed to explore for 30 s, and followed by turning on the lighting with the gate raised. The gate was lowered after the mouse models moved toward the dark region of the box and allowed to stay for 30 s before being physically removed and returned to their respective housings. After 30 min in their respective housings, the respective mouse models were transferred back into the box, and they were subjected to an electric shock in the foot (0.5 mA for 10 s) every time they entered the dark component. On the probing day (day 2-day 3), the respective mouse models were placed in the light component and left to explore for 270 s; with the following process similar to those outlined in day 1, with the absence of electric foot shock. This process was continued for the entire 1.5 months duration and any changes was noted accordingly [49,50,51,52].

##### Immunohistochemistry and Brain Tissue Section

To assess the formation and property (IgG2b expression) of the hybridoma cell growing environment in the MTAMs, the investigation of the timeline was prolonged to 2 months. Each mice mouse was carefully anesthetized with xylazine/zoletil mixture (1:1) diluted 10 times by ddH_2_O via IP and followed by transcardial perfusion with 0.9% NaCl (normal saline). The respective mouse models were sacrificed according to the approved procedure of IACUC/IACUP of Taipei Medical University. Next, the mice brain tissues were dissected into 2 halves and one of the halves was immersed into 10% neutral buffered formalin for 1 day at 4 °C. After fixation, the brain samples were sent to the CIS-biotechnology (Taichung, Taiwan) to proceed with paraffin-embedded, slicing into histological slides where briefly, paraffin-embedded brain tissue, the brain tissues were dehydrated by immersing them in gradually increasing concentration of ethanol (70–100%) and pure xylene. The dehydrated tissues were embedded in a 60 °C melted paraffin. The brain tissues sections were cut with thickness of 4 µm by microtome before being stored at room temperature. When immunohistochemistry staining is ready to be carried out, tissue slides were deparaffinized and rehydrated by first in xylene and gradually diluted ethanol (95–50%). The tissue slides were placed in a plastic rack with antigen-retrieval buffer (CIS-BIO, D3316). The rack was transferred into a 95 °C water bath for 10 min and was left to cool. The respective slides were washed with 0.025% Triton X-100 in Tris-Buffered Saline (TBS) (Tris Base, NaCl and pH 7.6). Next, slides were blocked in serum and 1% BSA in TBS for 2 h. at room temperature (RT), then washed with 0.025% Triton X-100 in TBS. After the tissue slides were treated with 0.3% H_2_O_2_ in TBS for 15 min to inactivate endogenous peroxidase activity, which caused high background, Dako REAL™ EnVision™ Detection System (EnVision) secondary antibody (K5007) (Labeled Polymer) was applied for 30 min. The tissue slides were then rinsed with TBS. The IgG2b expression was visualized by adding the substrate, Dako REAL™ EnVision™ Detection System (DAB) for 3 min (2 times). Before visualizing, the tissue slides were counterstained by Hematoxylin-Eosin (H&E) Staining Kit-for paraffin sections (CIS biotechnology M700, Taipei, Taiwan). When this step was completed, tissue slides were air-dried and applied Leica micro mount, REF(3801731) with the coverslip. In the case of phosphorylated tau, the procedure was quite similar to mentioned above. Briefly, after antigen-retrieval with 10 mM sodium citrate (pH 6.0), washed and blocking, Tau (phosphor Ser199/Ser202) primary antibody (GeneTex, GTX24864, Taipei, Taiwan) was added with a dilution of 1:20 in 1% BSA in TBS, then incubated at 4 °C overnight. The subsequent procedure was mentioned above. The HRP-conjugate 2nd antibody was added and incubated for 1 h. at RT. At last, the samples waited for chromogen (DAB) incubation for 10 min at RT. For counterstaining, the same kit was used. The coverslip tissue slides were then scanned by Tissue Gnostics Advancing Tissue Cytometry and analyzed by Strata Quest Tissue Analysis Solutions and ImageJ.

##### Western Blot

Fresh sample of brain tissues from the mouse models were snap freeze and store at −80 °C before being used. For protein extraction, prepare tissue lysate samples by adding tissue lysis buffer (RIPA buffer) (0.05 M Tris, pH 8.0; 0.005 M EDTA, 0.15 M NaCl, 0.5% SDS, 1% Triton 100X + 1 tablet of protease inhibitor), 1-time freeze and thaw, and 3-time centrifugation at 4 °C at 15,000 rpm for 15 min. The resulting supernatant was collected, and the protein quantification was carried out via Bradford assay. The entire unit was assembled accordingly with the ladder (4–20% Tris-glycine SDS PAGE) and the entire test was carried out at 100 volts for 1 h. Next, the gel was placed against the PVDF membrane, and sandwiched between filter papers and sponges where the electrophoretic transfer to the membrane at 100 voltages at cool temperature (0–4 °C) for 1hr. After completing the electrophoretic transfer, the membrane was blocked with a blocking buffer (3–5% BSA in TBST or 5% milk) to avoid the non-specific binding sites, followed by washing with TBST (0.05% Tween 20 in Tris buffer, pH 7.4) for 3 times (each time 10 min); and incubated with primary antibody (Anti-Tau (phosphor Thr205) antibody (GTX24841)) and Beta-actin (GTX629630) at 4 °C overnight. Next, incubation with the secondary (Goat anti-rabbit IgG (HRP), GeneTex and goat anti-mouse IgG (HRP)) was carried out (preceded by a 3× wash with TBST), at ambient conditions for 1 h. The membrane was reacted with an ECL kit (Western Lightning ECL Pro, Enhanced Chemiluminescence Substrate, P. Intertrade Equipment Co., Ltd., Khlongsan Bangkok, Thailand) by mixing 2 reagents in a 1:1 ratio. Finally, the signal (Chemiluminescence) was detected by Image Quant™ LAS 4000 (GE Healthcare, Chicago, IL, USA).

## 5. Conclusions

The novel PSF MTAMs with its unique microstructure did indeed perform well as a platform for encapsulated cell therapy that can maintain long term IgG2b antibody releasing hybridoma cells. The implantation of this platform indeed served as a proof-of-concept of an encapsulated cell therapy that is capable of positively impacting the outcome of the progression of Alzheimer’s disease. With future developments and adaptation to changing regulations, among the noteworthy ones-the cell source allogenic versus xenogeneic; potentially this technology could serve as a potential solution for this challenging disease.

## Data Availability

Not applicable.

## Supplementary Materials

The following supporting information can be downloaded at: https://www.mdpi.com/article/10.3390/ijms23126855/s1.
