# Supplementary material for: Microtube Array Membrane Encapsulated Cell Therapy: A Novel Platform Technology Solution for Treatment of Alzheimer’s Disease
Source: Int J Mol Sci. 2022 Jun 20;23(12):6855. doi: 10.3390/ijms23126855 (PMC9224941; doi:10.3390/ijms23126855)
Supplement: Supplementary file 1 [file ijms-23-06855-s001.zip › ijms-1749511-supplementary.pdf]

Supplementary data

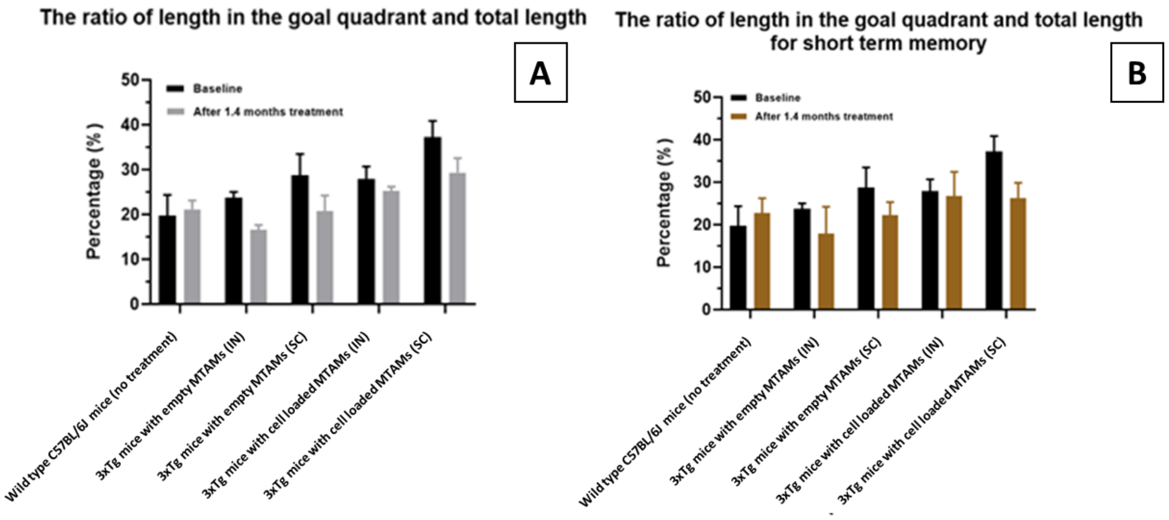

Figure S1. (A & B) The percentage of the ratio of length in goal quardrant (duration) and total length (total duration) of long-term memory and short-term memory assessment which revealed identical travel times in goal quadrant between the ratio of the long term memory (E) and those of the short term memory (F), thereby implying a consistent relationship between the two.

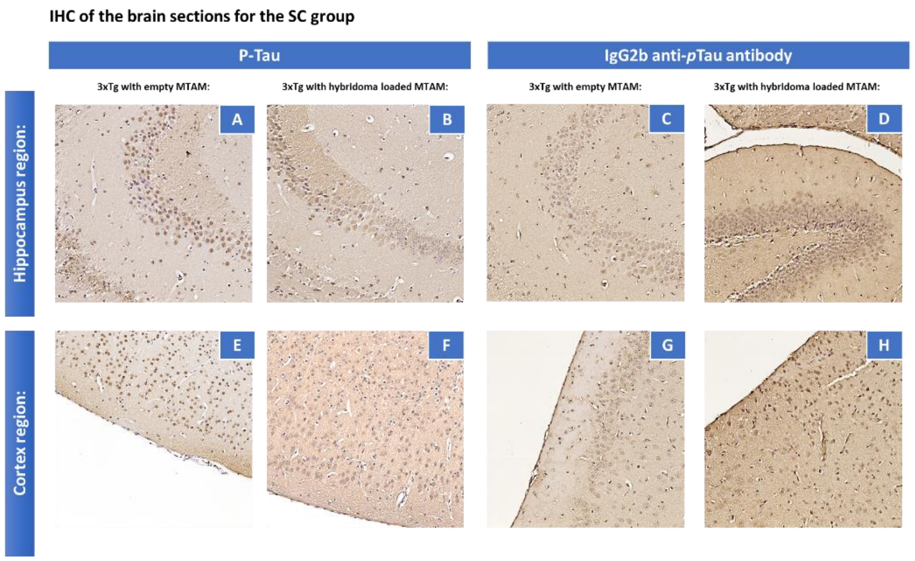

Figure S2. (A & C) Brain section of the 3xTg mice with empty MTAM of the Hippocampus region; and (E & F) of the similar study group of the cortex region. (B & D) 3xTg mice with the hyrbidoma cell loaded implanted of the hippocampus region; and (F & H) of the cortex region.
